# Thank You to Our Peer Reviewers!

**Published:** 2015-11-01

**Authors:** The Editors of JADPRO

In this, the final 2015 issue of the *Journal of the Advanced Practitioner in Oncology* (JADPRO), we would like to acknowledge all of the esteemed professionals who completed manuscript peer reviews for JADPRO in the past year. As a group, you represent a staggering depth and breadth of experience and expertise in the hematology/oncology field. You provide consistent high-quality, insightful, and rigorous critiques of submitted manuscripts, along with unerring professionalism and good humor.

We hope that during the past year we have fully given you our thanks and made our appreciation to you clear. Your contributions, largely done behind the scenes, have been indispensable. We hope that publishing the names of all of the 2015 JADPRO peer reviewers will be another step toward giving you the recognition you deserve for your incredible work. We couldn’t do it without you!

If you’d like to be a peer reviewer for JADPRO, contact claudine@harborsidepress.com for more information about how to get involved.

All the best for health and happiness in 2016!

—From the Editors of JADPRO

Paula Anastasia, RN, MN, AOCN®

Bradley Atkinson, PharmD

Catherine S. Bishop, DNP, NP, AOCNP®

Tami Borneman, RN, MSN, CMS

Ingrid Bowser, MS, APRN, ADM-BC, AOCNP®

Jeannine M. Brant, PhD, APRN, AOCN®

Jackie Broadway-Duren, FNP

Christopher J. Campen, PharmD, BCPS, BCOP

Kevin W. Chamberlin, PharmD

Mary Caroline Deigert, BS, MPAS, PA-C

Hollie Devine, MSN, RN, ANP-BC, AOCNP®

Morgane Diven, PharmD, BCOP

Raj Duggal, PharmD, BCOP

Beth Eaby-Sandy, MSN, CRNP, OCN®

Denice Economou, RN, MN, CNS, AOCN®, CHPN

Beth Faiman, PhD, MSN, APN-BC, AOCN®

Janice Famorca-Tran, RN, MS, AOCNP®

Regina Fink, RN, PhD, AOCN®, FAAN

Erica Glass, CRNP, MS, RN, BSN, AOCNP®

Amy Goodrich, MSN, CRNP

Carolyn Grande, CRNP, AOCNP®

Marilyn Haas-Haseman, PhD, ANP-BC

Jacqueline Hale, RN, APN, AOCN®

Susan Hawbaker, MSN, APN, ANP-BC, OCN®

Megan J. Holder, APN

Patrick M. Horne, MSN, ARNP, FNP-BC

Heather M. Hylton, MS, PA-C

Lisa Kottschade, RN, MSN, CNP

Kristen Kreamer, CRNP, MSN, AOCN®, APRN, BC

Sandra E. Kurtin, RN, MS, AOCN®, ANP-C

Gail Kwarciany, RN, MSN, BC, OCN®, AOCNS®

Joanne Lester, CRNP, ANP-BC, AOCN®

Julianne Leuck, MS, FNP-BC, APNP

Lydia T. Madsen, PharmD, RN, AOCNS®

Ashley Martin, ANP-BC

Karon Martyn, MSN, ANP-BC, AOCNP®

Kelley D. Mayden, MSN, FNP, AOCNP®

Erin McMenamin, MSN, CRNP, ANP-BC, AOCN®

Lynora Metoyer, RN, MSN, FNP

Susan Newton, RN, MS, AOCN®, AOCNS®

Nancy M. Nix, PharmD, BCPS, BCOP

Patricia Palmer, RN, MS, AOCNS®

Lisa Parks, MS, RN, CNP, ANP-BC

Jody Pelusi, PhD, FNP, AOCNP®

Todd Pickard, MMSC, PA-C

Julie Ponto, PhD, RN, ACNS-BC, AOCN®

Karen Roesser, RN, MS, AOCN®

Gayle Roux, PhD, NP-C, FAAN

Leanne M. Sakamoto, PharmD, BCOP

Julie Scott, ANP-BC

Lisa Sheldon, PhD

Wendy J. Smith, RN, MSN, ACNP, AOCN®

Robin M. Sommers, DNP, ANP-BC, AOCNP®

Mady Stovall, RN, MSN, ANP-BC

Virginia Sun, RN, PhD

Eric D. Tetzlaff, MHS, PA-C

Linda Thomson, PhD, APRN, ABMH

Sara Tinsley, ARNP, MS, AOCN®

Carol S. Viele, RN, MS, CNS, OCN®

Constance Visovsky, PhD, RN, ACNP-BC

Wendy H. Vogel, MSN, FNP, AOCNP®

Julie Walker, MSN, RN, FNP-C

Dwanna Ward-Boahen, DNP, NP-C, AOCNP®

Steven H. Wei, MS, MPH, PA-C

Rita Wickham, PhD, RN, AOCN®

Susan Wojcicki, PharmD, BCOP

James Young, PharmD

Jennifer Zadlo, PharmD, BCOP

Laura Zitella, MS, RN, ACNP-BC, AOCN®

